# Association of 25‐hydroxyvitamin D status with brain volume changes

**DOI:** 10.1002/fsn3.2382

**Published:** 2021-06-15

**Authors:** Yeonjae (Angel) Lee, Sungjin Yoon, SangYun Kim, Young Chul Youn

**Affiliations:** ^1^ Department of Psychological and Brain Sciences Johns Hopkins University Baltimore MD USA; ^2^ Chung‐Ang University College of Medicine Seoul Korea; ^3^ Deparment of Neurology Seoul National University College of Medicine and Seoul National University Bundang Hospital Seongnam Gyeonggi‐do Korea; ^4^ Department of Neurology and Department of Medical Informatics Chung‐Ang University College of Medicine Seoul Korea

**Keywords:** Alzheimer's disease, brain volume, cognitive impairment, vitamin D, voxel‐based morphometry

## Abstract

Vitamin D is critical to brain function and its deficiency accelerates cognitive impairment. There is limited understanding of the brain‐specific areas that undergo volume change in relation to blood vitamin D levels. The objective of this study was to evaluate the association between blood 25‐hydroxyvitamin D (25(OH)D) concentration and structural changes in the brain. We analyzed structural three‐dimensional T1 MRI images of 201 elderly individuals (mean age = 74.91 ± 9.21 years; 68.1% female; mean 25(OH)D = 18.05 nmol/L), with 10 community‐based normal healthy subjects, 33 with subjective cognitive decline, 97 with mild cognitive impairment, and 61 with Alzheimer's disease (AD). To analyze the structural changes in the brain respective to blood 25(OH)D, multiple regression analyses were performed using voxel‐based morphometry with age and total intracranial volume as covariates. Lower 25(OH)D level were associated with reduced brain volume in right olfactory, rectus GM regions (FWE‐corr, *p* < .05) for entire subjects. For AD subjects, left parahippocampal, fusiform, and hippocampal areas were positively associated with 25(OH)D (FWE‐corr, *p* < .05). Low blood 25(OH)D was associated with reduced volumes in olfactory and hippocampal regions in elderly patients with cognitive decline. Our results may provide insight into the neurological pathophysiology of vitamin D.

## Graphical abstract

This study evaluates the association between blood vitamin D concentration and structural changes in the brain. We found that lower vitamin D level was associated with reduced brain volume in right olfactory, rectus gray matter regions for entire subjects and left parahippocampal, fusiform, and hippocampus areas for subjects with Alzheimer's disease.

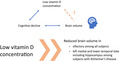

## 1 INTRODUCTION

Vitamin D is known to be critical to brain function and has neuroprotective effects (Anjum et al., 2018). Low levels of blood vitamin D accelerate cognitive impairment, and it especially results in executive dysfunction and episodic memory impairment (Miller et al., 2015). This is known to happen through regulating the release of neurotrophic factors, increasing antioxidant capacity, and decreasing the production of inflammation markers (Eyles et al., 2005; Grant, 2009; Moore et al., 2005). The high density of vitamin D receptors (VDR) in the hippocampus, hypothalamus, thalamus, cortex, and substantia nigra suggests the potential of vitamin D to influence neurological conditions (Fleet, 2004). Vitamin D contributes to neuronal development by regulating the synthesis of nerve growth factor (NGF) and various neurotransmitters such as acetylcholine (Ach), dopamine (DA), and gamma‐aminobutyric acid (GABA) (Moretti et al., 2017). Participants with lower vitamin D levels had worse performance on cognitive tests and slower information processing speed (Byrn & Sheean, 2019). Numerous cross‐sectional studies have proven the correlation between vitamin D and cognitive state (Chai et al., 2019; Schlögl & Holick, 2014). Low vitamin D level was found to be associated with low scores in cognitive tests such as MMSE or MoCA (Sultan et al., 2020). On the other hand, participants with higher vitamin D concentrations were less likely to develop cognitive impairment and related neurological diseases such as Alzheimer's disease (Feart et al., 2017; Goodwill et al., 2018). One study showed that their vitamin D supplementation group seemed to have lower brain amyloid‐beta (Aβ) levels, as evidenced by their higher plasma Aβ levels when compared to the placebo group (Miller et al., 2016). In another study, a group given high doses of vitamin D (from 67.2 ± 20 to 130.6 ± 26 nmol/L) improved their performance in nonverbal visual memory tasks as compared to a group given low doses of vitamin D (60.5 ± 22 to 85.9 ± 16 nmol/L) (Pettersen, 2017). Although some cross‐sectional studies failed to find correlations between vitamin D levels and direct cognitive impairment, the negative association between vitamin D levels and the risk for neurological complications has been well established and still remains a plausible hypothesis. Randomized‐controlled trials and cohort studies are needed to further verify the casual link between these two factors (Anjum et al., 2018; Sultan et al., 2020).

In the elderly, brain atrophy in both regions of lateral ventricles and the whole brain (Annweiler, Annweiler, et al., 2014) has been associated with low vitamin D levels. Low vitamin D levels were also associated with increased risk of cognitive decline (Annweiler et al., 2015), and vitamin D supplementation was shown to reduce fall risk (Bischoff‐Ferrari et al., 2009). However, there is still limited understanding on which specific brain region hypovitaminosis D is associated with, and we examined MRI images to confirm the known associated regions and also identify potential regions of the brain that are specifically correlated with vitamin D levels.

## 2 MATERIALS AND METHODS

### 2.1 Research design and participants

The study data were from Chung‐Ang University Hospital Dementia Registry. The study was approved by the institutional review boards of the Chung‐Ang University Hospital (2009‐005‐19331). The data were collected from May 2018 to March 2020, and a total of 226 subjects satisfied the inclusion criteria and had serum 25‐hydroxyvitamin D (25(OH)D) measurement.

The inclusion criteria for community‐based healthy normal control (HNC) in the study were as follows: (a) absence of memory complaints, (b) normal general cognition (within 1 standard deviation (*SD*) of the age‐ and education‐adjusted norms of the Korean version of the MMSE and a score >26), (c) normal activities of daily living (ADL), (d) Korean Dementia Screening Questionnaire <7, and (e) absence of depression (short form geriatric depression score <8). Patients that met the following criteria were categorized as suffering from subjective cognitive decline (SCD): (a) presence of memory complaints; (b) normal general cognition (within 1 standard deviation (*SD*) of the age‐ and education‐adjusted norms of the Korean version of the MMSE (K‐MMSE), and a score of >26); (c) normal ADL; and (d) no abnormalities on a comprehensive neuropsychological battery (within 1 *SD* of age‐ and education‐adjusted norms). Mild cognitive impairment (MCI) was categorized by the following: (a) complaints of memory inadequacies; (b) normal ADL; (c) objective cognitive impairment in more than one cognitive domain on a comprehensive neuropsychological battery (at least 1.0 *SD* below age‐ and education‐adjusted norms); (d) CDR of 0.5; and (e) not demented according to the Diagnostic and Statistical Manual of Mental Disorders (DSM)‐IV criteria. The patients with Alzheimer's disease dementia (AD) qualified the probable AD criteria proposed by the National Institute of Neurological and Communicative Disorders and Stroke and AD and Related Disorders Association (NINCDS‐ADRDA), as well those proposed by the DSM‐IV.

**FIGURE 1:**
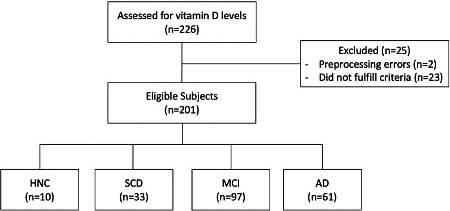
Enrollment of eligible subjects

Among 226 subjects, the subjects that were clinically diagnosed with Parkinson's disease or its variants, or other noncognitive neurodegenerative diseases were excluded. We also excluded two subjects whose nifti MRI images could not generate the 3D reconstruction of the brain. The number of final included subjects was 201. The subject demographics are as follows: 10 HNC, 33 SCD, 97 MCI, and 61 AD (Figure 1).

In this study, the serum 25(OH)D levels were referenced from the dementia registry database, which were measured using chemiluminescent immunoassay with using the ADVIA Centaur® Vitamin D Total Assay (Siemens Healthcare Diagnostics, Inc, NY) (Chen et al., 2012).

### 2.2 Preprocessing of MRI for VBM analysis

MRI scans in DICOM format were acquired from 3‐T scanners manufactured by Philips (Achieva). We conducted voxel‐based volumetry (VBM) to analyze our data using Computational Anatomy Toolbox (CAT12) in the most recent version of Statistical Parametric Mapping software (SPM12). CAT12 is an extension of Statistical Parametric Mapping software (Ashburner, 2012) created by the Structural Brain Mapping Group at the University of Jena to perform automated, quantitative analysis of the brain structures (Kurth et al., 2015). DICOM MRI files were converted into nifti files using MRIcron (http://people.cas.sc.edu/rorden/ mricron/index.html). Preprocessing of those nifti files were performed by CAT12 toolbox within SPM12, using “ICBM template‐East Asian Brains” template with default settings. All scans were normalized using an affine followed by nonlinear registration and were corrected for homogeneous bias. It was then segmented into GM, WM, and CSF. We used the Diffeomorphic Anatomic Registration Through Exponentiated Lie algebra algorithm (DARTEL) to normalize the segmented scans into a standard Montreal Neurological Institute space. Nonlinear deformation on the normalized segmented images was performed to correct individual brain size difference. We visually inspected the data after the automated procedures and excluded abnormal images that were not properly segmented or normalized. Rest of the segmented, modulated, and normalized GM and WM images were smoothed using 2‐mm full‐width‐half‐maximum Gaussian smoothing.

### 2.3 Statistical analysis

We analyzed the MRI files of the subjects using voxel‐based morphometry (VBM) to examine the regions of brain atrophy in cortical gray matter (GM) and white matter (WM) correlated with 25(OH)D levels. The smoothed images were analyzed via a linear multiple regression tool in SPM12 to detect negative correlation between vitamin D level and the GM and WM, respectively. Total intracranial volumes (TIV) were obtained through the “Estimate TIV” function in CAT12. In the matrix design, age and TIV were included as covariates to minimize potential confounding variables. The morphological difference in GM and WM was detected in p value (FWE‐corrected) of 0.05 with extent threshold at 30 voxels for total and 50 voxels for subgroups. We performed multiple regression analysis in all subjects, and in each subgroup.

## 3 RESULTS

### 3.1 Demographics of the subjects

The mean age of the total subjects was 74.91 ± 9.21 (mean ± standard deviation) years, and 138 were females and 64 were males. The mean 25(OH)D levels of the entire subjects were 18.05 ± 9.59. There was no significant statistical difference in the 25(OH)D levels between groups. The mean age of HNC was 70.4 ± 6.07 years, and 5 were females and 5 were males. The mean age of SCD was 69.81 ± 8.86 years, and 25 were females and 8 were males. The mean age of MCI was 75.62 ± 7.71 years, and 67 were females and 30 were males. The mean age of AD was 78.09 ± 8.36 years, and 40 were females and 21 were males (Table 1).

**TABLE 1:** Demographics of the subjects

	Number of subjects (female:male)	25(OH)D	Age
Sex			
Female	138	18.10 ± 9.74	74.29 ± 8.48
Male	64	17.94 ± 9.35	77.01 ± 8.31
Clinical state			
HNC	10 (5:5)	15.65 ± 6.35	70.4 ± 6.07
SCD	33 (25:8)	20.59 ± 10.20	69.81 ± 8.86
MCI	97 (67:30)	17.78 ± 9.86	75.62 ± 7.71*
AD	61 (40:21)	17.29 ± 9.31	78.09 ± 8.36*

Abbreviations: AD, Alzheimer's disease; HNC, healthy normal control; MCI, mild cognitive impairment; SCD, subjective cognitive decline.

* *p* < .05, a significance of the difference when compared with HNC by *t* test.

### 3.2 Correlation between GM/WM and Blood 25(OH)D

**FIGURE 2:**
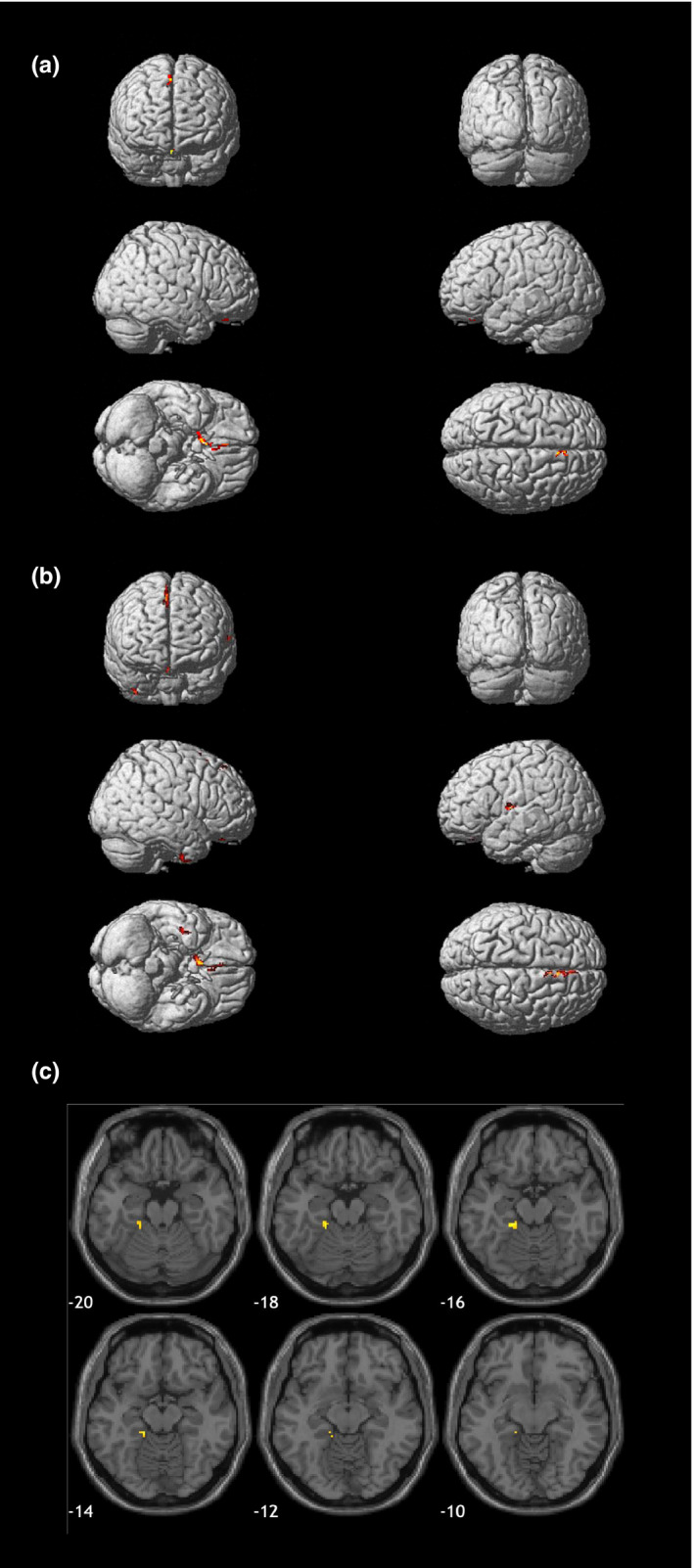
Low volume of gray matter regions positively associated with blood 25(OH)D level, corrected for age and total intracranial volume, family‐wise error (FWE) < 0.05 in total subjects (a), MCI subgroup (b), and AD subgroup (c)

The association between GM/WM and 25(OH)D level is shown in Table 2. There were no regions in WM that were correlated with 25(OH)D levels. When we analyzed the total 201 subjects, for GM, reduced right olfactory and rectus area were correlated with low 25(OH)D levels (Figure 2a). In SCD subjects, there was no significant correlation between 25(OH)D and total brain volume. For subjects with MCI, there was a positive association between 25(OH)D level and the following GM areas: right olfactory, rectus, supplementary motor area, left medial cingulate, superior temporal, and Rolandic operculum areas (Figure 2b). Left parahippocampal, fusiform, cerebellum, and hippocampus areas were also shown to be positively associated with 25(OH)D level in AD (Figure 2c).

**TABLE 2:** Anatomical labeling of brain structure negatively correlated to vitamin D level via Montreal Neurological Institute (MNI) coordinates

	MNI coordinate (*x*, *y*, *z*)	Label	*t* value	*z* value	*p* value (FEW‐corr)
Total	4, −14, 18	Olfactory_R Rectus_R	4.51	4.4	.008
MCI	4, −14, 18	Olfactory_R Rectus_R	5.11	4.78	.002
	−10, −18, 45	Cingulate_Mid_L	4.97	4.66	.033
	6, 21, 58	Supp_Motor_Area_R	4.93	4.64	.001
	−56, −6, 9	Rolandic_Oper_L Temporal_Sup_L	4.64	4.39	.044
AD	−21, −34, −15	Parahippocampal_L Fusiform_L Cerebellum_L Hippocampus_L	5.06	4.58	.012

Note

*p* value of maximal intensity cluster by voxel size is represented.

Abbreviations: Inf, inferior; L, left; Mid, middle; Oper, operculum; R, right; Sup, superior.

## 4 DISCUSSION

Preclinical evidence supports vitamin D playing a role in brain health and morphology. Vitamin D appears to assist calcium homeostasis in neurons during cerebral development by regulating the synthesis of calcium‐related cytoplasmic proteins such as parvalbumine or calbinding protein, and thereby reducing the expression of calcium channels (Annweiler et al., 2010). In another study, vitamin D administration prevented apoptosis and amyloid‐β peptide toxicity in neurons by increasing vitamin D receptor activity and downregulating calcium channels (Dursun et al., 2011). Moreover, studies have shown vitamin D plays a life‐long role in supporting essential brain functions and its deficiency has been linked to neurological and psychiatric disorders (Annweiler et al., 2010). Previous research had found hippocampal volume reduction in a serum 25(OH)D deficient group (<12 ng/ml) (Al‐Amin et al., 2019), as well as a thinner cingulate cortex, reduced whole brain volume, and a larger lateral cerebral ventricle (Annweiler et al., 2013; Buell et al., 2010; Foucault et al., 2019). This is in line with our finding that the lack of vitamin D is associated with reduced brain volume. Although there has been little focus on identifying focal brain atrophy in relation to vitamin D levels, recent study found that lower volume of the left calcarine sulcus was associated with lower serum 25(OH)D concentration (Ali et al., 2020). Our study provides additional information on the structural changes in brain volume related to vitamin D levels.

Our study demonstrated that a decrease in the subjects' overall vitamin D levels was associated with brain volume reduction of olfactory function‐related areas. Some studies have noted a link between hypovitaminosis D and a diminished sense of smell (Bigman, 2020; Shin et al., 2020), which was found to be ameliorated by increasing serum vitamin D (Kruse & Cambron, 2011). Another study found that low serum vitamin D concentrations were independently associated with olfactory dysfunction among Parkinson's disease patients (Kim et al., 2018). Olfactory impairment is associated with incident amnestic MCI and progression from amnestic MCI to AD (Devanand et al., 2015; Roberts et al., 2016), and also correlates with the severity of dementia and many other neurodegenerative pathologies (Murphy et al., 1990; Wilson et al., 2007). Vitamin D may be a useful biomarker for screening AD‐related amnestic disorder (Woodward et al., 2017; Yu et al., 2015). Previous studies showed that vitamin D deficiency is negatively associated with brain health, as such deficiencies were found to be correlated with cognitive dysfunction and neuropsychiatric disorders like AD (Annweiler, Karras, et al., 2014; Dickens et al., 2011; Fan et al., 2020; Keeney & Butterfield, 2015; Littlejohns et al., 2014; Mayne & Burne, 2019; Taghizadeh et al., 2014). In our results from subjects with AD, the brain regions associated with the decrease in vitamin D levels included regions in the left medial and lower temporal lobe, which are related to the memory domain of cognitive function. Medial temporal atrophy affecting the amygdala and hippocampus is typically observed in AD. The AD brain has also other cortical atrophy that is most marked in multimodal association cortices: temporal and parietal, while primary motor and somatosensory cortices appear mostly unaffected (Perl, 2010). In other words, the more severe the vitamin D deficiency in our AD patients, the more severe the atrophy of the medial temporal lobe, including the hippocampus. These observations support the conclusion that vitamin D can act against neurodegenerative processes. Also, as the reduced regions are central to normal cognitive states (e.g., executive functions, memory consolidation, and behavioral regulations), cognitive impairment of people with low 25(OH)D levels may be explained by the brain atrophy of the regions that are responsible for neurocognitive functions.

The effects on medial cingulate, superior temporal, and Rolandic operculum areas found in MCI subgroup as well as parahippocampal, fusiform, cerebellum and hippocampus areas in AD subgroup were all found in the left hemisphere but not the right hemisphere. Previous MRI studies have demonstrated that regions that are vulnerable to atrophy in AD progression, particularly in the left hemisphere, are associated with a higher risk of developing dementia and memory impairments (Chételat et al., 2005; Galton et al., 2000; Querbes et al., 2009; Risacher et al., 2010). Because the left is the dominant hemisphere, cognitive symptoms caused by lesions in the hemisphere are readily detected clinically. Under the well‐established relationship between the clinical progression of AD symptoms and brain atrophy (Jack et al., 2010), our data suggest that the pathology of AD leading to neuronal loss may be related to vitamin D levels.

However, despite some promising results, there is still insufficient evidence that supplementation of vitamin D can treat AD or alleviate its symptoms (Landel et al., 2016). In our subjects with MCI, the brain volume reductions correlated with vitamin D levels were identified in areas that were not necessarily related to AD pathology. Since the diagnosis of MCI is made clinically, the causative disease may include various neurodegenerative diseases other than just AD, for example frontotemporal lobe degeneration, early‐stage Parkinson's disease, diffuse Lewy body disease, or cerebral small vessel disease, all of which can negatively impact cognitive function, especially frontal executive functions. Epidemiological and clinical studies found that reduced vitamin D level is associated with an increased risk of Alzheimer's disease, vascular dementia (Moretti et al., 2017; Wang et al., 2020), Parkinson's disease (Evatt et al., 2008, 2011; Lin et al., 2003; Wang et al., 2001), prion disease (Suenaga et al., 2013), and motor neuron disease (Blasco et al., 2015; Yang et al., 2016). The individuals in the MCI subgroup in this study likely suffer from various causative diseases that could have impacted various areas of brain volume reduction related to vitamin D levels. Furthermore, some regions that were found to be significantly correlated with vitamin D in MCI but not in AD could be attributed to the ceiling effect: there has already been such significant progression of brain atrophy in AD that vitamin D is no longer statistically correlated with the volume changes in the regions found in MCI. Rather, the medial temporal lobe, an area critical to the progression of AD, is found to be negatively associated with vitamin D levels in the AD subgroup.

One limitation of this study is the fact that it is a cross‐sectional study with one‐time measurement of vitamin D levels. Additionally, correlation analysis between vitamin D levels and cognitive tests was not conducted. Further longitudinal or follow‐up studies about the causal relationship between brain atrophy region and cognitive deficits would provide a better understanding of the pathology of vitamin D deficiency in cognitive impairment.

## CONFLICT OF INTEREST

The authors declare no conflict of interest.
